# Exploring the impact of artificial sweeteners on diabetes management and glycemic control

**DOI:** 10.3389/fnut.2025.1587690

**Published:** 2025-08-12

**Authors:** Rukaiah Fatma Begum, S. Nirenjen, Rapuru Rushendran, M. Manisha, N. Pavithra, S. Sridevi, S. Ankul Singh

**Affiliations:** ^1^Institute of Pharmaceutical Research, GLA University, Mathura, Uttar Pradesh, India; ^2^Department of Pharmacology, SRM College of Pharmacy, SRM Institute of Science and Technology, Chennai, Tamil Nadu, India; ^3^Department of Pharmacology, Faculty of Pharmacy, Dr. M.G.R Educational and Research Institute, Chennai, Tamil Nadu, India; ^4^Department of Pharmaceutical Analysis, Faculty of Pharmacy, Dr. M.G.R Educational and Research Institute, Chennai, Tamil Nadu, India; ^5^Department of Pharmacy Practice, National Institute of Pharmaceutical Education and Research (NIPER), Guwahati, Assam, India

**Keywords:** artificial sweetener, diabetes, glycemic control, blood glucose levels, insulin sensitivity

## Abstract

Diabetes mellitus (DM), a chronic metabolic disorder characterized by impaired glucose metabolism, has emerged as a significant global health challenge. Effective management of diabetes encompasses not only medical interventions but also lifestyle and dietary modifications. Artificial sweeteners (ASs), due to their low caloric content and minimal impact on blood glucose levels, offer promising potential as sugar substitutes for individuals aiming to manage glycemic control. Compounds such as aspartame, sucralose, and stevia mimic the sweetness of sugar without causing hyperglycemia, making them suitable for diabetic patients. This chapter explores the role of ASs in diabetes management, with a special focus on their mechanisms of action, including modulation of insulin sensitivity and glucose metabolism. An extensive review of preclinical and clinical studies evaluates the efficacy, safety, and long-term effects of ASs in glycemic control, highlighting their ability to reduce caloric intake, promote satiety, and support glycemic control and insulin sensitivity in individuals with diabetes. Emerging evidence suggests that ASs may influence gut microbiota, potentially affecting metabolic outcomes and insulin sensitivity, thus presenting opportunities for personalized interventions. Despite their benefits, awareness of potential risks, such as altered taste perception and over-reliance on ASs, is crucial. Integrating ASs within a broader lifestyle approach, comprising regular exercise and balanced nutrition, ensures optimal outcomes in diabetes management. This chapter emphasizes the importance of precision medicine in tailoring AS use to individual metabolic responses, underscoring its role as an adjunct to comprehensive diabetes care strategies.

## 1 Introduction

Diabetes mellitus (DM) is a chronic metabolic disorder characterized by persistently high blood sugar levels resulting from insufficient insulin production, insulin resistance, or both. Its global prevalence has quadrupled since 1990, now affecting over 800 million adults, making it a major health crisis (1). This dramatic rise is largely fueled by aging populations, increasingly sedentary lifestyles, and the growing prevalence of obesity, which contributes to the rising incidence of type 2 diabetes (T2D). Alarmingly, more than half of all diabetes cases occur in low- and middle-income countries, where access to essential healthcare and treatments remains a significant challenge (2). Diabetes can lead to severe complications such as heart disease, kidney failure, neuropathy, vision loss, and amputations, contributing to significant global morbidity and mortality. In 2021, it was linked to ~6.7 million deaths worldwide (3). Beyond its devastating health impact, diabetes also carries a heavy financial toll. In 2021, global healthcare spending related to diabetes reached an estimated US$966 billion, a staggering 316% increase over the past 15 years (4). Effective blood sugar control is vital to prevent complications and improve the quality of life in individuals with diabetes. This requires lifestyle changes, regular monitoring, and medical care. However, many remain undiagnosed or untreated, highlighting the need for increased awareness and accessible healthcare (5, 6).

Managing diabetes effectively requires a well-rounded approach that combines lifestyle changes with proper diagnostic approaches (7) and medical treatments (8). Healthy daily habits, especially dietary choices, are essential for regulating blood sugar levels and maintaining overall wellbeing. Carbohydrate intake directly affects glucose levels, and organizations like the ADA emphasize the importance of mindful eating for effective diabetes control (9). Managing diabetes involves choosing low-glycemic foods, controlling portion sizes, and reducing added sugars. A balanced diet with fiber, lean proteins, and healthy fats supports blood sugar control and insulin sensitivity. Regular physical activity further enhances insulin efficiency and glucose uptake (10). Maintaining an active lifestyle also supports weight management, which is especially important for people with type 2 diabetes, as excess weight is often linked to insulin resistance (11). In addition to diet and exercise, habits like regular meal timing, blood sugar monitoring, and stress management help stabilize glucose levels. Stress can increase blood sugar, so relaxation and sleep become important. Combined with medication, these lifestyle changes enhance treatment effectiveness and reduce the risk of complications (12, 13).

With increasing efforts to reduce sugar intake, Artificial sweeteners (ASs), such as aspartame, sucralose, saccharin, and stevia, offer low-calorie alternatives found in many foods and drinks. For people with diabetes, they provide sweetness without spiking blood sugar, aiding in glucose control and complication prevention (14, 15). Despite their popularity, ASs remain under scrutiny for possible effects on metabolism, insulin sensitivity, and appetite. Some may trigger insulin responses or alter gut bacteria, potentially impacting digestion, inflammation, and blood sugar regulation (16). To understand ASs' role in diabetes management, it is crucial to consider their benefits, risks, and health impacts. They can help reduce sugar intake and manage blood sugar levels, but individual responses vary, and potential side effects should not be ignored. Ongoing research is essential to understand the long-term effects better and establish clear guidelines for both people with diabetes and the general population (17). This manuscript aims to critically evaluate the current evidence on the impact of ASs on glycemic control and their potential role, favorable or adverse, in the management of diabetes mellitus.

## 2 Mechanisms of action of ASs

### 2.1 Mimicking sweetness without causing hyperglycemia

Consuming sugary foods and drinks increases the risk of obesity, a key contributor to insulin resistance and the onset of type 2 diabetes, metabolic syndrome, and heart diseases, which are closely linked to diabetes. Reducing sugar intake is crucial for managing blood sugar and preventing complications. While natural sweeteners may help lower blood sugar and improve metabolism, the long-term safety of AS remains under debate and requires further research (18). Innovative strategies for low-sugar, low-fat beverages aim to maintain taste, texture, and appearance while supporting glycemic control in diabetes management. These approaches use natural sugar and fat replacers, with future efforts focusing on developing new replacers, enhancing sensory profiles, and investigating their health impacts (19). A study found that chronic consumption of ASs at safe intake levels caused vascular endothelial dysfunction and increased adipose tissue storage in healthy rats, potentially impairing insulin sensitivity and glycemic control. This suggests a link to the elevated cardiometabolic risk seen in epidemiological studies (20). Aspartame has been shown to exacerbate obesity, inflammation, and gut dysregulation—factors closely linked to insulin resistance and diabetes pathophysiology. This suggests that *Siraitia grosvenorii* extracts could serve as a promising natural alternative to ASs in preventing metabolic disorders, including insulin resistance and type 2 diabetes (21). The ability of meals to increase blood sugar levels after a meal is ranked using the glycemic index (GI). Patients with diabetes should eat foods with a low GI since controlling blood sugar levels is crucial. It is well recognized that eating foods with a high GI can cause rapid, high, and persistent postprandial hyperglycemia, which makes it more challenging to manage diabetes and avoid complications (22, 23). Zùsto^®^ is a low-glycemic index sweetener (GI 22) that may help manage glycemic control in diabetic patients. It has better consumer acceptance than other ASs due to its lack of aftertaste and minimal effect on insulin and C-peptide levels. Its potential long-term benefits, including reduced glycemic variability, warrant further studies to confirm its efficacy (24). *Stevia rebaudiana*, a natural sweetener with zero calories, is rich in steviol glycosides like stevioside and rebaudioside (25). These compounds offer promising health benefits, such as antimicrobial, antiobesity, anticancer, and antidiabetic properties (26). Stevia is a safe, non-toxic alternative to ASs, used in foods, drinks, and supplements (27).

### 2.2 Impact on glycemic load and blood glucose levels

ASs affect blood glucose levels differently, depending on their type and use. Studies show Stevia and nano-Stevia may help manage diabetes and its complications. Stevia refers to the crude or conventional extract of the Stevia plant, containing natural glycosides used as sweeteners (28). In contrast, nano-Stevia is a nanoformulation of Stevia, in which Stevia compounds are encapsulated or processed at the nanoscale to enhance their bioavailability, stability, and efficacy. This nanosizing is believed to improve absorption and facilitate improved therapeutic outcomes compared to regular stevia extracts (29). In diabetic rats, both Stevia and nano-Stevia reduced hyperglycemia, anxiety, and memory issues, with nano-Stevia proving more effective, suggesting its potential in treating diabetes-related metabolic and psychological disorders (30). Another study highlighted the beneficial effects of stevioside in improving glucose tolerance, reducing oxidative stress, and modulating inflammatory mediators in a diet-induced obese zebrafish model. These effects suggest that stevioside may counteract insulin resistance through epigenetic, oxidative stress, and inflammatory regulation, providing insights into its potential as a natural therapeutic agent for obesity-related type 2 diabetes by preventing the development of insulin resistance, which is a key driver of disease progression (31). Other ASs, like the stevia derivative Reb M, have been studied for their effects on metabolic health. Long-term use of the majority of ASs does not cause weight gain or impaired glucose metabolism in mice. Reb M, in particular, improved insulin sensitivity and reduced weight gain in obese mice, emphasizing the need to evaluate each AS individually (32, 33). The effects of saccharin on glucose metabolism depend on the context. Short-term studies indicate that it is safe for healthy individuals, but some findings suggest it may disrupt glucose balance in certain conditions (34). High-dose saccharin does not affect gut microbiota or induce glucose intolerance in healthy humans or mice, indicating that it is likely safe when consumed within recommended limits for those managing weight or calorie intake (35). A study found that saccharin disrupted glucose balance in rats and reduced glucagon-like peptide-1 (GLP-1) release during glucose tolerance tests, even though insulin secretion remained unchanged. This may be due to saccharin interfering with the link between sweet taste and caloric intake, potentially leading to hyperglycemia, increased food intake, and weight gain (36). These findings underscore the potential metabolic risks of ASs, aligning with human data linking their use to obesity and adverse health outcomes.

### 2.3 Interaction with sweet taste receptors (STRs) and signaling pathways

AS interacts with sweet taste receptors, affecting signaling pathways involved in glycemic control and metabolism. Early-life consumption of ASs can impact taste preferences, sugar-related behaviors, and metabolic outcomes, as seen in rodent studies (37). Studies suggest that ASs may affect the gustatory system, influencing both sweetness and glucose-sensing pathways. This could have long-term effects on sugar intake and metabolism, potentially contributing to metabolic diseases such as diabetes. Additionally, sweet and bitter tastants can trigger the release of satiety hormones, such as cholecystokinin (CCK) and GLP-1, with some commercial sweeteners, such as Tagatesse (a blend containing polyols and sweeteners), being reported to be more effective than sucrose in stimulating hormone release, indicating their potential to regulate appetite (38). These findings highlight the role of taste stimuli in regulating satiety and food intake, which can aid in developing strategies for managing appetite and diabetes. One study explored the crystal structure of Mabinlin II (Mab II), a sweet protein from *Capparis masaikai*. The human sweet taste receptor is a heterodimer composed of two G protein-coupled receptor subunits, hT1R2 and hT1R3. This receptor complex is primarily expressed in taste bud cells on the tongue. It is responsible for detecting sweet-tasting compounds, including natural sugars (like glucose and sucrose), AS (such as aspartame and sucralose), and certain sweet proteins. Upon activation by a sweet stimulus, hT1R2/T1R3 initiates a signaling cascade that leads to the perception of sweetness in the brain. Beyond the oral cavity, this receptor is also expressed in other tissues, including the gut and pancreas, where it may play roles in nutrient sensing and metabolic regulation. Sweetness perception in humans is mediated by the heterodimeric sweet-taste receptor T1R2/T1R3, which is expressed on the surface of taste receptor cells within the taste buds (39). This receptor consists of two subunits, T1R2 and T1R3, that together form a binding site capable of recognizing a wide variety of sweet compounds (40). The unique structural features of [the compound or sweetener], such as its specific arrangement of functional groups, allow it to bind to the T1R2/T1R3 receptor with high affinity. This binding activates intracellular signaling pathways that ultimately trigger the sensation of sweetness. Its unique structure enables it to interact with the sweet-taste receptor hT1R2/T1R3, triggering the perception of sweetness. The B-chain of Mab II is responsible for sweetness, while the A-chain contributes to a lasting aftertaste. These insights could lead to the development of new sweeteners based on Mab II's structure (41). Fibroblast growth factor 21 (FGF21) is a hormone that is induced by sugar and plays a critical role in regulating sweet consumption. Elevated plasma levels of FGF21 are associated with reduced sweet preference, suggesting its role in regulating sugar intake by influencing the brain's reward system (42). Research has shown that FGF21 interacts with glutamatergic neurons in the ventromedial hypothalamus (VMH), where it suppresses sucrose intake by increasing neuronal activation. Notably, the induction of FGF21 is triggered by ingestion of simple sugars, including sucrose, rather than AS. This highlights its role in maintaining metabolic balance and nutrient regulation. Understanding FGF21′s mechanisms could help develop therapies to address unhealthy eating habits and manage diabetes (43). Steviol glycosides, such as stevioside and rebaudioside A, enhance the TRPM5 channel in pancreatic β-cells and taste receptor cells. This improves taste perception and increases insulin secretion in response to glucose. This mechanism helps prevent diabetic hyperglycemia in mice, making TRPM5 a promising target for affordable and effective diabetes treatments (44). The T1R2 receptor functions as a glucose sensor in the intestine, facilitating the control of glucose absorption by regulating the movement of the GLUT2 transporter in enterocytes. Studies show that T1R2-mediated glucosensitization is important during high sugar intake, as it boosts glucose transport through GLP-2. However, long-term sugar consumption may lead to desensitization of this pathway, thereby preventing hyperglycemia as metabolic diseases progress (45).

## 3 Role in glucose regulation and insulin sensitivity

### 3.1 ASs and glucose absorption

ASs, though designed to provide sweetness without raising blood glucose levels, can influence glucose absorption in the gut through multiple mechanisms. Some ASs, like sucralose and saccharin, may activate sweet taste receptors (T1R2/T1R3) on intestinal cells, leading to increased expression of glucose transporters such as SGLT1 and GLUT2, which can enhance glucose uptake when sugar is present (46). Additionally, these sweeteners may alter the gut microbiota, potentially promoting inflammation and impairing glucose tolerance and insulin sensitivity (47). While the direct effects on glucose absorption in humans remain inconsistent across studies, the influence of AS on the gut environment suggests they are not metabolically inert. Notably, stevia appears to have a more neutral or even beneficial profile, possibly improving insulin sensitivity without significantly affecting glucose absorption (48).

ASs, including intense sweeteners such as saccharin, aspartame, and acesulfame-K (ace-K), and a bulk sweetener like glucose, exert different effects on hunger and food intake (49). Glucose reduces hunger and affects food preferences, while intense sweeteners may increase hunger and preferences but slightly reduce meal intake due to sensory stimulation (49, 50). Aspartame has the strongest effect, showing the complex role of sensory, cognitive, and post-ingestive factors in appetite control. ASs, like sucralose and saccharin, may impact glycemic responses in healthy adults by altering the microbiome and metabolome (34). These effects vary by individual, suggesting ASs can cause microbiome-dependent changes in glycemic control, requiring more research (51). Caloric sweeteners such as glucose and fructose inhibit motilin secretion and antral motility while increasing CCK secretion, which promotes satiety. In contrast, the AS acesulfame-K (ace-K) does not affect these gastrointestinal processes, suggesting that caloric and AS influence gastrointestinal motility and hormone secretion differently, potentially impacting hunger regulation (52). Further research is needed to explore ASs that mimic the effects of caloric sweeteners on appetite control. The Diabetes Research in Kids Type 1 Diabetes (DRINK-T1D) study examines the effect of the AS restriction on glycemic variability, adiposity, lipid profiles, and inflammation in children with type 1 diabetes. The study suggests that ASs could offer cardiometabolic benefits, prompting a revision of current nutritional guidelines for children with T1D (53). The active compound 3-hydroxymethyl xylitol (3-HMX), isolated from the root of Casearia esculenta, demonstrates significant long-term antihyperglycemic effects in streptozotocin-diabetic rats by enhancing insulin secretion and inhibiting gluconeogenesis, exhibiting comparable efficacy to glibenclamide while remaining non-toxic to hepatic enzymes (54). Another study reveals that 77.8% of Chilean pregnant women consumed ASs daily, with sucralose being the most prevalent. Its consumption was significantly associated with an increased risk of gestational diabetes mellitus, highlighting a pressing need for further research and dietary considerations for pregnant populations (55).

However, research also demonstrates that ruminant intestines express T1R2-T1R3, which can be activated by ASs like Sucram to enhance glucose absorption and intestinal growth (56). Activation of these receptors increases GLP-2 release, enhancing SGLT1 expression and mucosal development, and suggesting dietary strategies to improve nutrient absorption in ruminants. Additionally, adding 60 g of fructose as a natural sweetener to the diet of obese type II diabetes patients for 12 weeks may slightly improve glycemic control without harming lipid metabolism (57). These results suggest alterations in apoprotein composition that might reduce the risk of coronary artery disease, with no significant adverse effects observed.

Health professionals are advised against adding sweeteners to foods for infants and young children (1–3 years) and are encouraged to develop expertise in selecting suitable sweeteners. Further research is needed to optimize their use in children's diets (58). Regular consumption of ASs, such as saccharin, sucralose, or aspartame + acesulfame-K, for 4 weeks did not significantly affect glycemic response, insulin sensitivity, GLP-1 secretion, or body weight in healthy individuals (59). These findings suggest that ASs intake at typical daily doses is metabolically neutral in normoglycemic adults. However, one crossover trial found that acute saccharin consumption did not significantly affect glycemic response or insulin levels in healthy young men, although minor, non-significant increases in mean insulin levels were observed after saccharin intake compared to water (60). This highlights the need for larger, long-term trials to clarify saccharin's metabolic effects across populations. Meanwhile, stevia leaf extract, a natural sweetener, significantly enhances SGLT1 activity and expression in rabbits, improving glucose absorption and reducing *Escherichia coli*-induced diarrhea (61).

### 3.2 Influence on insulin secretion and sensitivity

ASs, including aspartame, have varied effects on glucose regulation and insulin sensitivity. Aspartame was detected in saliva at higher levels after consuming diet soft drinks compared to water with sweeteners, and increased salivary insulin levels were observed, particularly with diet soft drinks, showing a correlation between salivary aspartame and insulin levels (62). These findings suggest potential biological effects and health implications of AS consumption. However, a 12-week study indicated that consuming two cans daily of a carbonated beverage containing aspartame and acesulfame-K had no significant impact on insulin sensitivity or secretion in nondiabetic adults (63). High-intensity sweeteners in beverages do not affect key metabolic markers, such as insulin levels, body weight, or behaviors. However, sucrose intake during a mixed meal does not cause additional hyperglycemia compared to isocaloric starch in well-controlled diabetic patients, suggesting that moderate consumption of sucrose, a nutritive sweetener, may be compatible with good glycemic management (64). Similarly, studies on saccharin consumption in Wistar rats revealed weight gain without an increase in total caloric intake or associations with insulin resistance, fasting leptin, or peptide YY (PYY) levels (65). Saccharin's sweet taste may contribute to weight gain by affecting glucose transport or energy expenditure, and further research is required. Stevioside from *Stevia rebaudiana* shows significant antihyperglycemic, insulin-promoting, and glucagon-inhibiting effects in type 2 diabetic rats, highlighting its potential as a novel antidiabetic agent (66). Furthermore, beverages sweetened with sucrose (a caloric sugar), sucralose, or stevia showed varied effects on glycemic responses and insulin sensitivity (65, 67–71). At the same time, stevia demonstrated slightly better glycemic control, whereas sucrose and sucralose impaired glycemic response and insulin sensitivity. However, the evidence remains inconclusive to support the widespread use of ASs (72). In healthy adults, inhibition of gastrointestinal sweet taste receptors with lactisole—a compound known to block sweet taste perception —was found to modify insulin responses during oral glucose intake, leading to elevated plasma insulin levels and inducing mild, temporary insulin resistance (73). These findings suggest that STR-mediated mechanisms in the gut may regulate glycemia, possibly through gut–brain axis interactions, requiring further research. Additionally, sugar-sweetened beverages (SSBs) are associated with increased insulin resistance, higher insulin levels, and elevated leptin levels, particularly in men and non-overweight women (74). These biomarkers—such as elevated fasting insulin, C-reactive protein, and markers of hepatic fat accumulation—suggest that metabolic dysfunction can arise early in response to the consumption of SSBs. This effect is particularly evident even among women with normal BMI and waist circumference, indicating that excess sugar intake can trigger insulin resistance, low-grade inflammation, and ectopic fat deposition independently of overt obesity. These pathophysiological changes are well-established precursors to type 2 diabetes and other metabolic disorders. This revision clarifies the mechanistic link between these biomarkers and disease risk.

### 3.3 Comparative analysis of common ASs (e.g., aspartame, sucralose, and stevia)

Maternal consumption of artificial sweeteners, particularly acesulfame K (ace-K), is associated with adverse metabolic outcomes in mice, including glucose intolerance, metabolic dysfunction, and fetal growth restriction (75). Similarly, sugar-sweetened beverages (SSBs) have been shown to impair maternal glucose metabolism, promote excessive gestational weight gain, and increase the risk of adverse pregnancy outcomes such as gestational diabetes mellitus and macrosomia (76). Both ASs and SSBs have been linked to shortened pregnancy duration and altered fetal glucose levels, raising concerns about their safety during pregnancy (77). High consumption of refined carbohydrates and sugars is strongly implicated in the rising rates of obesity and diabetes, underscoring the need for effective strategies to reduce their intake. These dietary habits induce oxidative stress and β-cell damage in genetically predisposed individuals, contributing to the onset and progression of metabolic diseases (78). Some ASs, such as stevia and aspartame, show promise in diabetes management. Preloads with stevia or aspartame do not increase food intake compared to sucrose, and stevia has been shown to reduce postprandial glucose and insulin levels, indicating potential benefits for glucose regulation and weight management (79). Natural alternatives, such as miracle fruit (*Synsepalum dulcificum*), show potent antihyperglycemic and hepatoprotective properties. Its ethanol extract, rich in flavonoids and antioxidants, effectively reduces blood glucose and restores liver function in diabetic models, outperforming aspartame, suggesting its potential as a natural substitute for ASs (80). However, not all sugar substitutes yield positive results. Long-term consumption of sorbitol has been shown to alter the gut microbiome, reducing the abundance of beneficial bacteria, such as *Bifidobacterium* and *Lachnospiraceae*. These changes contribute to glucose intolerance and may increase the risk of diabetes (81). Similarly, artificially sweetened beverages (ASBs) and SSBs are associated with an increased risk of diabetes mellitus (DM), particularly in postmenopausal women. SSBs pose a higher risk (43%) compared to ASBs (21%) (82). While substituting sugar-sweetened or artificially sweetened beverages with water significantly reduces diabetes risk, further research and innovation are needed to make sweetened drinks healthier, as complete substitution with water may not be feasible or acceptable for all consumers.

## 4 ASs and the gut–brain axis

**Figure 1:**
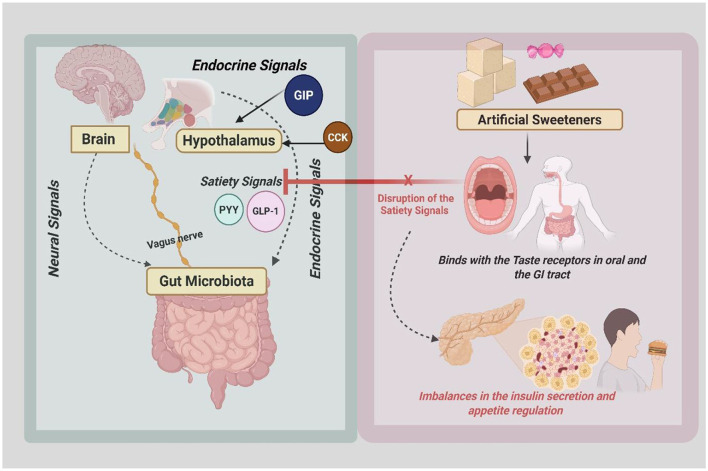
Role of gut–brain axis and ASs in diabetes. CCK, cholecystokinin; GIP, gastric inhibitory polypeptide; PYY, peptide YY; GLP-1, glucagon-like peptide-1.

The gut–brain axis (GBA) is a complex communication network connecting the brain's emotional and cognitive centers with intestinal functions (Figure 1). This relationship is bidirectional, with signals traveling through hormonal, immune, and neural pathways. When food is consumed, the gut sends signals to the brain, conveying information about the meal's composition and size. The hypothalamus, located between the thalamus and pituitary gland in the brain, acts as a key regulator by processing these signals to control energy use, food intake, and glucose balance. Special cells in the gut, called enteroendocrine cells, detect the contents of the gut and release hormones such as cholecystokinin and glucagon-like peptide-1 to help manage metabolism (83, 84).

According to the National Health and Nutrition Examination Survey (NHANES), a program conducted by the Centers for Disease Control and Prevention designed to assess the health and nutritional status of adults and children in the United States through interviews and physical examinations, the prevalence of AS consumption increased from 6.1 to 12.5% in the younger population and from 18.7 to 24.1% in the adult population between 1999 and 2007. While more recent cycles of the NHANES have been conducted, comprehensive published analyses quantifying AS consumption trends beyond 2007 remain limited, underscoring the need for updated surveillance (85). Other statistical data, such as the Nurses' Health Study (56% usage reported between 1991 and 1995), the Health Professionals Follow-Up Study (54% usage between 1990 and 1994), and the San Antonio Heart Study (48% usage between 1984 and 1988), reported even higher usage rates over different periods (86). Despite the increasing use of ASs, the consumption of sugary beverages and foods has not decreased (87). The mechanisms through which AS influence appetite and satiety are rooted in their effects on behavioral and neurochemical pathways. Sugar is known to have a high potential for addiction due to its ability to bind opioid receptors, and downstream effects on dopamine and acetylcholine release in the nucleus accumbens. These neural adaptations promote the reward phenomenon, which leads to excessive sugar consumption. Food reward is regulated through both sensory and post-ingestion pathways (88). These altered reward mechanisms may drive excessive sugar intake, which can worsen glycemic control and contribute to the development of type 2 diabetes.

ASs influence satiety and appetite regulation through intricate mechanisms involving post-ingestion pathways and appetite regulation. When AS are consumed, they in turn activate sweet taste receptors such as T1R2 and T1R3 in the gastrointestinal tract and oral cavity (89). These receptors send signals to the reward centers of the brain, including the amygdala and hypothalamus, via neural pathways such as the vagus nerve. This signaling triggers the release of dopamine and generates the perception of sweetness in areas associated with pleasure and reward. However, unlike sugar, ASs provide no caloric energy, leading to a mismatch between the absence of an expected rise in blood glucose levels and the sensory perception of sweetness and insulin secretion (90). This disruption creates an imbalance in physiological satiety signals, leaving the body in a state of incomplete fullness, which can disrupt glycemic control in individuals with diabetes. Additionally, AS may alter the release of key gut hormones involved in appetite regulation, such as glucagon-like peptide-1 (GLP-1), peptide YY (PYY), and ghrelin (91). These disruptions can impair the natural cues for fullness and hunger. Neurochemical pathways play a critical role as well, where AS overstimulates dopamine release without the accompanying caloric intake, leading to desensitization of the reward system and requiring higher levels of sweetness or increased food consumption to achieve the same level of satisfaction (90). Modulation of other neurotransmitters, such as acetylcholine and opioid pathways, further complicates the regulation of appetite and satiety. The overall outcome of these mechanisms is an increase in food cravings and appetite, often resulting in higher caloric intake (15, 92, 93).

## 5 Impact of ASs on gut microbiota composition

A study utilized the fecal samples from healthy volunteers, and the study demonstrated that additives such as aspartame-based sweetener, maltodextrin, and sodium benzoate promoted the growth of beneficial microbes such as *Bifidobacterium*, whereas substances like carrageenan-kappa, sodium sulfite, and polysorbate-80 inhibited their growth (94). These additives and products also affected the production of short-chain fatty acids (SCFAs), with acetic acid levels increasing with maltodextrin and aspartame-based sweeteners but decreasing with sodium sulfite. In contrast, butyrate levels, a vital SCFA for gut health, dropped significantly with cinnamaldehyde.

Furthermore, microbial diversity was altered, with stevia increasing α-diversity and cinnamaldehyde (94). Another study highlighted the profound effects of ASs on the structure and functionality of duodenal microbial communities, emphasizing their potential to disrupt the delicate balance of gut health. The research reveals that ASs significantly reshape the microbial composition in the duodenum, the critical first segment of the small intestine where digestion and nutrient absorption begin (95). ASs can disrupt gut microbiota diversity and function, especially in the duodenum, potentially affecting gut health and metabolism. With the rising consumption of ASs, these findings highlight the need for further research into their long-term health effects and interactions with the gut microbiome (95).

Changes in gut microbiota are now recognized as key to understanding how metabolic disorders, such as type 2 diabetes, develop. The gut microbiome plays a crucial role in maintaining metabolic balance, and an imbalance (dysbiosis) is closely linked to poor glucose control, insulin resistance, and T2DM (96). One significant mechanism involves increased intestinal permeability, often referred to as a leaky gut, which facilitates the translocation of bacterial components, like lipopolysaccharides, into the systemic circulation (97). This triggers chronic low-grade inflammation, a hallmark of metabolic syndrome. Dysbiosis also affects the production of short-chain fatty acids such as butyrate, acetate, and propionate, which are derived from the fermentation of dietary fibers. SCFAs influence energy metabolism, improve insulin sensitivity, and stimulate the secretion of gut hormones like glucagon-like peptide-1 and peptide YY (PYY), which regulate glucose levels and appetite (98, 99). A reduction in butyrate-producing bacteria, commonly observed in individuals with type 2 diabetes mellitus, is linked to decreased colonocyte health and impaired glucose homeostasis. Colonocytes are the epithelial cells that line the colon and rely on butyrate as a primary energy source to maintain gut barrier integrity, regulate inflammation, and support metabolic functions. Consequently, a decline in butyrate production may compromise intestinal health and contribute to dysregulated glucose metabolism (100). Additionally, the gut microbiota significantly impacts bile acid metabolism, converting primary bile acids into secondary forms that regulate glucose metabolism through receptors, such as farnesoid X receptor (FXR) and TGR5 (101). In individuals with type 2 diabetes, alterations in bile acids can interfere with key signals that help regulate blood sugar levels. Gut bacteria also affect levels of branched-chain amino acids (BCAAs), which, when elevated, are associated with insulin resistance. An imbalanced gut microbiome can raise BCAA levels and worsen glucose control. Targeting the gut microbiome may offer new treatment options (102, 103). For instance, supplementation with specific probiotics has been shown to increase GLP-1 levels and improve glucose tolerance in both animal models and human studies (104). Moreover, fecal microbiota transplantation from healthy donors to individuals with metabolic syndrome has resulted in improved insulin sensitivity, highlighting the causal role of gut microbiota in glucose metabolism (105). These findings underscore the intricate interplay between the gut microbiome and host metabolic processes, suggesting that restoring microbial balance could be a cornerstone in managing T2DM and related conditions. Dysbiosis promotes inflammation, disrupts energy metabolism, and impairs glucose regulation. Targeting the gut microbiome through diet, probiotics, or fecal transplants may reduce insulin resistance and improve metabolic health, offering the potential for managing and preventing T2DM and related conditions, including Alzheimer's disease (106, 107).

## 6 Preclinical and clinical studies

### 6.1 Summary of preclinical findings on ASs and diabetes

ASs have been widely investigated for diabetes management through studies ranging from animal models to clinical trials, offering insights into their efficacy, safety, and long-term effects. Preclinical studies in rodents, such as Sprague-Dawley rats and C57BL/6 mice, have shown mixed, dose- and context-dependent impacts on glucose metabolism. Notably, saccharin impaired glucose tolerance in C57BL/6 mice by altering the gut microbiota, thereby increasing *Bacteroides* populations associated with metabolic changes (34). This research highlighted the indirect impact of ASs on glucose regulation through microbial pathways, raising concerns about their broader metabolic effects. Further research used Zucker diabetic fatty (ZDF) rats to investigate the effect of sucralose on insulin sensitivity. Their findings showed that lower doses of sucralose improved insulin signaling by modulating hepatic glucose production. However, higher doses disrupted insulin pathways, illustrating the complexity of dose-dependent effects (108). Similarly, a study found that ASs induced significant changes in the gut microbiota, such as a reduction in *Lactobacillus* spp., which was linked to impaired glucose tolerance (109). This reinforced the idea that gut microbiota plays a crucial role in mediating the metabolic effects of ASs. In contrast, another study observed that saccharin-induced changes in microbial populations—particularly an increase in *Akkermansia muciniphila—*could affect glucose regulation and inflammatory pathways (110). These findings suggest that targeting the gut microbiota through dietary interventions may help mitigate some adverse effects of Ass and offer potential therapeutic strategies. Additionally, preclinical studies have examined the impact of ASs on weight management, demonstrating that aspartame reduced calorie intake and promoted weight loss in diet-induced obese (DIO) mice, likely through hypothalamic pathways involved in satiety, which may improve insulin sensitivity and glycemic control (111).

### 6.2 Clinical trials: safety and efficacy

Clinical trials are essential in evaluating the real-world applicability of AS for individuals with diabetes. These studies assess their impact on blood glucose control, insulin response, weight management, and overall safety profiles, providing crucial evidence for their use in diabetes management. Clinical studies suggest that AS does not raise blood glucose levels, making it a safe alternative to sugar for people with diabetes. For instance, a study involved 200 participants with type 2 diabetes and showed that replacing sucrose with aspartame over 12 weeks significantly reduced HbA1c levels without adverse effects (112). This finding underscores the potential of ASs in supporting glycemic control while minimizing caloric intake. Similarly, a review found that ASs like sucralose have negligible effects on both fasting blood glucose and postprandial glucose responses (113). While ASs generally do not directly influence insulin secretion, some studies suggest that certain sweeteners may trigger a response known as the cephalic-phase insulin release. For example, a study examined the effects of sucralose on insulin response in 40 non-diabetic individuals and found a slight, transient increase in insulin levels, though without significant metabolic consequences (114). Further studies suggest that the cephalic-phase insulin response induced by AS may vary among individuals and does not necessarily affect metabolic outcomes universally (115). ASs are also commonly recommended for weight management due to their low caloric content. The CHOICE study demonstrated that overweight participants who replaced sugar-sweetened beverages with AS-based drinks lost an average of 2.5 kg over 6 months (116). These results highlight the potential of ASs in reducing caloric intake when used in conjunction with lifestyle interventions. Additionally, a study noted that ASs might reduce cravings for sugary foods, further supporting their role in weight management strategies (117).

Long-term safety evaluations of AS indicate that they are generally well-tolerated. However, some studies raise concerns about gastrointestinal and metabolic effects. For instance, a study reported mild gut dysbiosis (an imbalance in gut bacteria) and gastrointestinal discomfort among high AS consumers in a cohort study of 500 participants (118). These findings are consistent with those of Suez et al. (34), who demonstrated that prolonged AS use can alter gut microbiota in susceptible individuals, potentially affecting glucose metabolism. Such studies emphasize the need for long-term follow-up trials to better understand the broader implications of chronic AS consumption (34). Overall, while AS provides significant benefits in terms of glycemic control and weight management, its long-term effects on gut health and overall metabolic outcomes require further investigation. Personalized approaches to incorporating AS into diabetes management plans should be considered, taking into account individual metabolic responses and potential risks associated with AS.

### 6.3 Long-term effects and risk-benefit analysis

Although ASs have become an integral component of dietary strategies aimed at managing diabetes, their long-term effects and risk-benefit profiles continue to spark debate. This discussion examines the nuanced outcomes of chronic AS use, focusing on critical areas such as chronic disease risk, gut microbiota implications, behavioral effects, and the importance of individualized guidance. Epidemiological studies raise concerns about the potential association between high AS consumption and chronic diseases, which are comorbid with or precursors to diabetes mellitus. For instance, a longitudinal study involving 1,000 individuals over 5 years found that saccharin use was linked to a slight increase in cardiovascular risk markers, such as elevated triglycerides and LDL cholesterol levels (119). However, because factors such as diet, exercise, and existing health issues may influence the results, more controlled studies are needed to confirm if saccharin directly causes these health risks. Long-term use of ASs has also been shown to impact gut microbiota, a key player in metabolic health. It is demonstrated that prolonged saccharin consumption in both animal models and humans led to dysbiosis, characterized by altered microbial diversity and an increase in pro-inflammatory species (34). These changes were associated with impaired glucose tolerance and decreased insulin sensitivity. Further studies highlighted that the metabolic implications of microbiota shifts depend on the individual's baseline gut composition and genetic predispositions (120). The psychological and behavioral effects of ASs play a significant role in their risk-benefit profile.

Sweeteners such as aspartame and sucralose may reinforce sweet preferences, potentially leading to overconsumption of sweet-tasting foods and beverages. Behavioral intervention studies suggest that educating patients on mindful AS consumption and understanding compensatory eating behaviors can mitigate these risks. For example, incorporating ASs into a balanced diet, rather than using them as unrestricted replacements for sugary foods, helps maintain overall caloric balance (50). The risk-benefit profile of ASs varies widely among individuals due to differences in health conditions, dietary habits, and metabolic responses.

## 7 Optimization of ASs for diabetes management

Effective utilization of ASs in diabetes management involves integrating them into patient care to maximize benefits and minimize risks. Strategies include substituting ASs for high-calorie sugars in beverages and food products, which can help reduce overall caloric intake and manage blood glucose levels (121). Clinical guidelines emphasize educating patients on the types and quantities of ASs that can be safely incorporated into their diet, focusing on balance and moderation (122). Combining ASs with broader lifestyle management programs, including regular exercise and a healthy diet, can enhance their efficacy in achieving glycemic control and weight loss (123). Emerging research on the gut microbiota's role in metabolic health presents opportunities for tailoring AS use based on individual microbiota profiles. A study also indicates that certain AS can induce shifts in gut microbial populations, potentially affecting glucose metabolism and insulin sensitivity (34). Personalized approaches, such as microbiota profiling, could identify individuals more likely to benefit from specific ASs while avoiding adverse effects like dysbiosis (124). This precision medicine approach ensures ASs are optimized to align with individual metabolic responses, offering a novel avenue for improving diabetes outcomes. Patient education on ASs is crucial in diabetes care, emphasizing both their benefits, such as lower calorie intake and improved blood sugar control, and potential risks, including changes in gut microbiota and long-term metabolic effects. Awareness efforts should discourage overuse, as it may lead to overeating or taste changes that can disrupt overall dietary balance (125). Health professionals should promote the mindful use of ASs as part of a broader diabetes management plan, ensuring intake stays within safe limits, such as the 40 mg/kg ADI for aspartame. Regular monitoring of glucose, HbA1c, and metabolic markers helps assess effectiveness and avoid potential harm, supporting personalized, evidence-based care (126).

## 8 Risks and challenges

ASs pose potential risks, including effects on insulin sensitivity, glucose metabolism, and gut microbiota, which may lead to inflammation and metabolic issues. Although calorie-free, some can trigger insulin responses or increase cravings, possibly leading to overeating and poor glycemic control in people with or at risk for diabetes. Although short-term studies indicate that ASs are mostly safe, their long-term effects on heart health and the development of chronic diseases remain uncertain. Confusing regulations and misleading labels make it difficult for consumers to make informed choices. Groups such as pregnant women, children, and those with metabolic issues may be more at risk; therefore, cautious use is advised (127).

### 8.1 Potential adverse effects of long-term AS use

Although ASs are commonly used as sugar substitutes, their long-term health effects are still debated. Some studies link regular use to metabolic imbalances, insulin resistance, and appetite disruptions (128). Another ongoing debate is whether ASs affect appetite and food intake. Some studies suggest that they may disrupt natural hunger signals, leading to stronger cravings for sweets and higher calorie intake, which can potentially result in weight gain. Although often marketed as beneficial for people with diabetes, some extensive studies have linked their use to higher risks of heart disease, metabolic syndrome, and even death. However, more research is needed to understand these links and the underlying mechanisms (129, 130).

### 8.2 Considerations for vulnerable populations

Health organizations such as the FDA and EFSA consider sweeteners like aspartame and sucralose safe within recommended limits, but saccharin is discouraged due to concerns about fetal exposure (131). Children are increasingly consuming ASs found in sugar-free snacks, drinks, and processed foods. As their metabolic systems are still developing, excessive intake could impact their insulin response, gut microbiota, and taste preferences (132). Experts worry that early exposure to ASs may lead to a preference for sweet foods, contributing to unhealthy eating habits and a higher risk of obesity and metabolic disorders later on. While some sweeteners are approved for children, many health organizations advise limiting their use, especially in younger children, until more research clarifies their long-term effects (129).

### 8.3 Ethical and regulatory aspects of AS consumption

The increasing use of ASs raises concerns about consumer safety, transparency, and public health, as well as ethical and regulatory issues. Agencies, such as the FDA, EFSA, and WHO, have established acceptable daily intake (ADI) levels based on current research; however, regulations vary by country, resulting in inconsistencies in approval and use. As scientific knowledge of ASs evolves, it is important to regularly update safety guidelines to reflect new findings (18). Moreover, ASs are currently common in processed foods and beverages, often marketed as “sugar-free” or “diet” products. However, many consumers may not fully understand their potential risks and benefits. Ethical concerns arise when food manufacturers fail to provide clear labeling or use marketing tactics that create misleading perceptions about their health effects. Regulatory agencies stress the importance of transparent food labeling and honest communication about ASs to empower consumers (133). The food and beverage industry greatly influences public perception of ASs, but critics argue that industry-funded research often highlights benefits while downplaying risks. Public health initiatives should provide balanced, evidence-based information to help people make informed dietary choices (134). Ethical concerns also involve vulnerable communities with limited access to fresh, natural foods. It is important to ensure that ASs do not disproportionately affect these groups, emphasizing the need for policies that prioritize safety and fair access to nutritious food (135).

## 9 Future directions and research opportunities

As ASs continue to be important in managing diabetes and reducing sugar intake, research is shifting toward exploring their broader metabolic effects, safety, and potential benefits beyond blood sugar control. Future directions in sweetener development focus on improving taste, safety, and effectiveness, with the integration of new technologies and personalized nutrition helping to create more suitable alternatives for people with diabetes. These innovations aim to provide sweetness without the negative effects of traditional sweeteners while supporting better metabolic health and insulin sensitivity. Researchers are also working on making sweeteners more stable and acceptable in various food products, enabling their successful integration into everyday diets.

Research opportunities in this field include further investigating the long-term effects of new sweeteners on insulin response, weight management, and metabolic health. As some sweeteners may affect gut microbiota, future studies should focus on understanding how these changes impact diabetes outcomes. Additionally, exploring the prebiotic effects of certain sweeteners, like stevia derivatives and allulose, and their role in gut health could offer promising therapeutic strategies. A deeper understanding of how sweeteners interact with hunger hormones and appetite regulation will also be crucial to refining their use. By addressing these gaps, researchers can pave the way for more personalized, effective dietary strategies for managing diabetes and improving overall metabolic health.

## 10 Conclusion

ASs are commonly used as sugar substitutes, offering a way to enjoy sweetness without significantly affecting blood glucose levels. They are especially useful in diabetes management when combined with personalized strategies that account for gut health and insulin dynamics, helping people control blood sugar and reduce calorie intake. Some sweeteners, such as allulose and stevia, may even improve insulin sensitivity. However, concerns persist about their long-term effects on insulin response, appetite, gut microbiota, and overall metabolism. Evidence linking ASs to changes in gut microbiota calls for further research to achieve a better understanding of their impact on glucose metabolism and inflammation. As demand for healthier sugar alternatives grows, future research should focus on creating new sweeteners that reduce metabolic risks while maintaining taste and stability in food. Personalized nutrition, based on individual variations in gut health and metabolism, could optimize sweetener use for diabetes management. It is also important to have transparent labeling and consumer education to ensure informed decisions. While ASs offer benefits for blood sugar control and weight management, they should not be seen as a one-size-fits-all solution. A balanced approach, incorporating whole foods and ongoing research, will be key to the effective and safe use in diabetes care.
